# Two Paralogous Tetraspanins TSP-12 and TSP-14 Function with the ADAM10 Metalloprotease SUP-17 to Promote BMP Signaling in *Caenorhabditis elegans*

**DOI:** 10.1371/journal.pgen.1006568

**Published:** 2017-01-09

**Authors:** Lin Wang, Zhiyu Liu, Herong Shi, Jun Liu

**Affiliations:** Department of Molecular Biology and Genetics, Cornell University, Ithaca, New York, United States of America; University of California San Diego, UNITED STATES

## Abstract

The highly conserved bone morphogenetic protein (BMP) signaling pathway regulates many developmental and homeostatic processes. While the core components of the BMP pathway have been well studied, much research is needed for understanding the mechanisms involved in the precise spatiotemporal control of BMP signaling *in vivo*. Here, we provide evidence that two paralogous and evolutionarily conserved tetraspanins, TSP-12 and TSP-14, function redundantly to promote BMP signaling in *C*. *elegans*. We further show that the ADAM10 (a disintegrin and metalloprotease 10) ortholog SUP-17 also functions to promote BMP signaling, and that TSP-12 can bind to and promote the cell surface localization of SUP-17. SUP-17/ADAM10 is known to be involved in the ligand-induced proteolytic processing of the Notch receptor. We have evidence that the function of SUP-17, and of TSP-12/TSP-14 in BMP signaling is independent of their roles in Notch signaling. Furthermore, presenilins, core components of the γ-secretase complex involved in processing Notch, do not appear to play a role in BMP signaling. These studies established a new role of the TSP-12/TSP-14/SUP-17 axis in regulating BMP signaling, in addition to their known function in the Notch signaling pathway. We also provide genetic evidence showing that a known BMP signaling modulator, UNC-40/neogenin/DCC, is one of the substrates of SUP-17/ADAM10 in the BMP signaling pathway.

## Author Summary

Bone morphogenetic protein (BMP) signaling regulates multiple developmental and homeostatic processes. Misregulation of this pathway can cause various diseases, including cancers. Thus, it is essential to understand how BMP signaling is tightly regulated spatiotemporally *in vivo*. We have identified a highly conserved ADAM (a disintegrin and metalloprotease) protein, SUP-17/ADAM10, as an important factor in modulating BMP signaling in *C*. *elegans*. We showed that the proper localization and function of this ADAM protease require two conserved tetraspanin proteins, TSP-12 and TSP-14. We provided genetic evidence showing that one of the substrates of SUP-17/ADAM10 in the BMP signaling pathway is a known BMP signaling modulator, UNC-40/neogenin/DCC. Our studies established a new role of the TSP-12-TSP-14-SUP-17 axis in regulating BMP signaling, in addition to and independent of their known function in the Notch signaling pathway.

## Introduction

The highly conserved bone morphogenetic protein (BMP) pathway is repeatedly used in metazoan development to regulate multiple distinct processes in different cellular contexts. In canonical BMP signaling, secreted BMP ligands bind to the heteromeric type I/type II receptor complexes and induce the phosphorylation of type I receptors by type II receptors, which in turn phosphorylate the R-Smads. The phosphorylated R-Smads then complex with Co-Smad and enter the nucleus to regulate gene transcription. Increasing evidence has shown that BMP signaling is tightly regulated spatiotemporally and that misregulation of this pathway can cause many different disorders in humans, such as cardiovascular diseases and cancers [1–5]. Recent studies have identified a number of factors that modulate BMP signaling at the level of the ligand-receptor complex [6–10]. In particular, we have previously shown that the small transmembrane tetraspanin proteins are important in promoting BMP signaling in *C*. *elegans* [7].

Tetraspanins are a large group of integral membrane proteins with a characteristic protein topology. They contain four transmembrane domains and a number of conserved residues, such as the featured CCG motif in the second extracellular loop [11]. Recent studies have shown that a subgroup of tetraspanins, the TspanC8 family of tetraspanins, functions to promote another highly conserved signaling pathway, the Notch pathway [12–15]. In particular, the TspanC8 tetraspanins physically associate with ADAM10 and promotes its cell surface localization. ADAM10, in turn, cleaves the Notch receptor in a process called “ectodomain shedding”, releasing the intracellular domain of Notch for subsequent activation of downstream gene expression [12–14]. Previously, we have shown that in *C*. *elegans*, the TspanC8 tetraspanins TSP-12 and TSP-14 function in modulating BMP signaling [7]. However, whether they exert their functions in BMP regulation by acting through the *C*. *elegans* ADAM10 protein is not known. Similarly, because of their roles in both BMP signaling and Notch signaling, it is not clear whether TSP-12 and TSP-14 regulate these two pathways independently. In this study, we investigated how TSP-12 and TSP-14 regulate BMP signaling in *C*. *elegans*.

**Fig 1:**
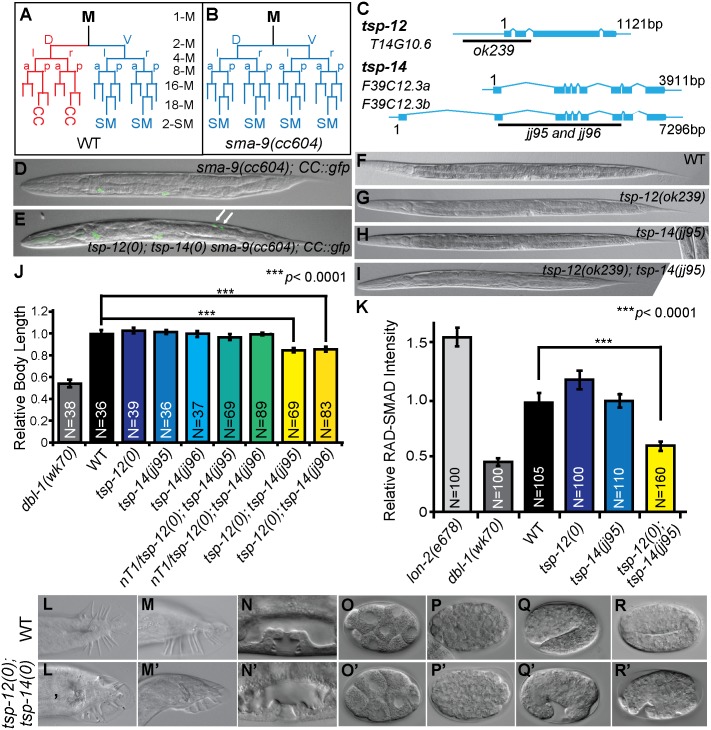
*tsp-12(0); tsp-14(0)* double mutants exhibit defects in Sma/Mab signaling. (A, B) Schematic representation of the M lineage in wild type (WT) or *tsp-12(0); tsp-14(0) sma-9(0)* (A), and *sma-9(0)* animals (B). CC: coelomocyte, SM: sex myoblast, v: ventral, d: dorsal, l: left, r: right, a: anterior, p: posterior. (C) Diagrams depicting the *tsp-12* and *tsp-14* genomic regions and the locations of *tsp-12(ok239)* and *tsp-14(jj95*, *jj96)* deletions. See also S1 Fig. (D, E) Merged GFP and DIC images of late L4 stage *sma-9(cc604)* and *tsp-12(ok239); tsp-14(jj95) sma-9(cc604)* worms carrying the coelomocyte (CC) GFP marker *CC*::*gfp*. Arrows point to M-derived CCs. (F-I) Representative DIC images of developmental stage-matched WT (F), *tsp-12(ok239)* (G), *tsp-14(jj95)* (H) and *tsp-12(ok239); tsp-14(jj95)* (I) worms, showing the smaller body size of *tsp-12(ok239); tsp-14(jj95)* worms. (J) Relative body length of developmental stage-matched worms of various genotypes grown at 20°C. The mean body length of WT worms is normalized to 1.0. Error bars represent 95% confidence intervals (CI) for the normalized body length. (K) Quantification of the RAD-SMAD reporter GFP fluorescence intensity in various mutants compared with WT animals (set to 1.0). Error bars represent 95% confidence intervals (CI) for the normalized mean RAD-SMAD fluorescence intensity. (L-R’) DIC images of WT (L, M, N, O, P, Q, R) and *tsp-12(ok239); tsp-14(jj95)* mutant (L’, M’, N’, O’, P’, Q’, R’) male tails (L-M), hermaphrodite vulva at the Christmas tree stage (N), and embryos at different developmental stages (O-R). (L’, M’, N’) Images of *tsp-12(ok239); tsp-14(jj95)* worms produced by heterozygous *tsp-12(ok239)/+; tsp-14(jj95)* mothers. (O’-R’) Images of *tsp-12(ok239); tsp-14(jj95)* embryos produced by homozygous *tsp-12(ok239); tsp-14(jj95)* mothers, showing ventral enclosure defects. See also S2 Movie.

The *C*. *elegans* BMP-like pathway is called the Sma/Mab pathway. This pathway is known to regulate body size, male tail patterning and mesoderm development [16]. Core members of this pathway include the ligand DBL-1/BMP, the type I and type II receptors, SMA-6/RI and DAF-4/RII, the R-Smads SMA-2 and SMA-3, as well as the Co-Smad SMA-4 [16]. Loss-of-function mutations in any of these core members will result in a small body size, male tail sensory ray formation defects [17], reduced expression of the RAD-SMAD reporter [18], and suppression of the *sma-9(cc604)* M lineage defect [19]. In particular, mutations in the *C*. *elegans schnurri* homolog *sma-9* cause a dorsal-to-ventral cell fate transformation in the postembryonic M lineage, thus the loss of two M-derived coelomocytes (CCs). Loss-of-function mutations in any core members of the Sma/Mab pathway can suppress this M lineage defect (Fig 1A and 1B), resulting in the reappearance of the two M-derived CCs. Thus, Sma/Mab pathway mutants exhibit a Susm (suppression of the *sm**a-9* M lineage) phenotype ([19]; Fig 1A and 1E). We have previously shown that the Susm phenotype can be used as a specific and sensitive assay to screen efficiently for mutants with defects in Sma/Mab signaling [7]. Using the Susm assay, we have identified several highly conserved factors that function to modulate BMP signaling at the level of the ligand-receptor complex. These include the RGM protein DRAG-1 [20], the neogenin/DCC homolog UNC-40 [18], the tetraspanin TSP-21 [7], as well as two redundant tetraspanins, TSP-12 and TSP-14.

Here, we show that TSP-12 and TSP-14 function redundantly in BMP signaling by regulating the cell surface localization of the ADAM10 (a disintegrin and metalloprotease 10) ortholog SUP-17. We demonstrate that the function of SUP-17 in BMP signaling is independent of its well-established role in Notch signaling. Finally, we provide genetic evidence indicating that a known BMP modulator, UNC-40/neogenin/DCC, is one of the substrates of SUP-17/ADAM10 in the BMP signaling pathway.

## Results

### The paralogous TSP-12 and TSP-14 function redundantly to promote BMP signaling

The *C*. *elegans* genome encodes 21 tetraspanins. We have previously reported a weak Susm (suppression of the *sm**a-9* M lineage) phenotype in the *tsp-12(ok239)* null mutants, which is enhanced by *tsp-14(RNAi)*, but not by RNAi of any of the remaining 19 *tsp* genes in *C*. *elegans* [7]. To further determine mechanistically how TSP-12 and TSP-14 function in Sma/Mab signaling, we generated three deletion alleles of *tsp-14* (*jj95*, *jj96 and jj97*) using the CRISPR/Cas9 system (Fig 1C; see Materials and Methods). We used both *jj95* and *jj96* in all our analyses described below, and found them to behave identically. We therefore refer to both alleles as *tsp-14(0)*. Similarly, we refer to the deletion allele *tsp-12(ok239)* as *tsp-12(0)*.

*tsp-12(0)* and *tsp-14(0)* single mutants are each fully viable and fertile. *tsp-12(0); tsp-14(0)* double mutants produced by *tsp-12(0)/+; tsp-14(0)* mothers are also viable, but exhibit vulva morphogenesis defect (Fig 1N’) and are egg-laying defective (Egl). They also exhibit multiple Sma/Mab signaling defects: unlike the *tsp-12(0)* or *tsp-14(0)* single mutants, these *tsp-12(0); tsp-14(0)* double mutants have a smaller body size (Fig 1F and 1J), exhibit reduced RAD-SMAD reporter activity (Fig 1K) and double mutant males have severe tail defects (Fig 1L’ and 1M’), including crumpled spicules, fused and shortened sensory rays, and smaller fans. *tsp-12(0); tsp-14(0)* double mutants also suppress the *sma-9(cc604)* M lineage defect at high penetrance (Fig 1A, 1B, 1D, 1E and Table 1). These observations indicate that TSP-12 and TSP-14 share redundant functions in promoting BMP signaling. In addition to these phenotypes, the *tsp-12(0); tsp-14(0)* double mutants also exhibit 100% maternal-effect embryonic lethality: all their eggs die in late embryogenesis, with defects in ventral enclosure (Fig 1O’ and 1R’ and SI Appendix, S1 and S2 Movies). The ability of *tsp-12(0); tsp-14(0)* double mutants produced by *tsp-12(0)/+; tsp-14(0)* mothers to survive through embryogenesis is likely due to the perdurance of maternally loaded *tsp-12* mRNA or TSP-12 protein by the *tsp-12(0)/+; tsp-14(0)* mothers. Thus, TSP-12 and TSP-14 share redundant functions that are essential for both embryonic and postembryonic development.

**Table 1:** TSP-12 and TSP-14 function redundantly to promote Sma/Mab signaling.

Genotype	Susm penetrance^a^ (# of animals examined)
*sma-9(cc604)*	—
*tsp-12(ok239); sma-9(cc604)*	13% (N = 343)
*tsp-14(jj95) sma-9(cc604)*	1% (N = 725)
*tsp-14(jj96) sma-9(cc604)*	0% (N = 829)
*nT1[qIs51]/tsp-12(ok239); tsp-14(jj95) sma-9(cc604)*	8% (N = 913)
***tsp-12(ok239); tsp-14(jj95) sma-9(cc604)***	73% (N = 405)*** ^b^
*nT1[qIs51]//tsp-12(ok239); tsp-14(jj96) sma-9(cc604)*	6% (N = 707)
***tsp-12(ok239); tsp-14(jj96) sma-9(cc604)***	64% (N = 591)*** ^b^

^**a**^ The Susm penetrance refers to the percent of animals with 1–2 M-derived CCs as scored by the *CC*::*gfp* reporter.

***p< 0.0001 (unpaired two-tailed Student’s *t*-test)

^**b**^ Statistical analysis was conducted by comparing the *tsp-12(ok239); tsp-14(0) sma-9(cc604)* triple mutants with the *tsp-12(ok239); sma-9(cc604)* double mutants.

### TSP-12 is widely expressed and is localized to both the cell surface and the cytoplasm in multiple cell types

**Fig 2:**
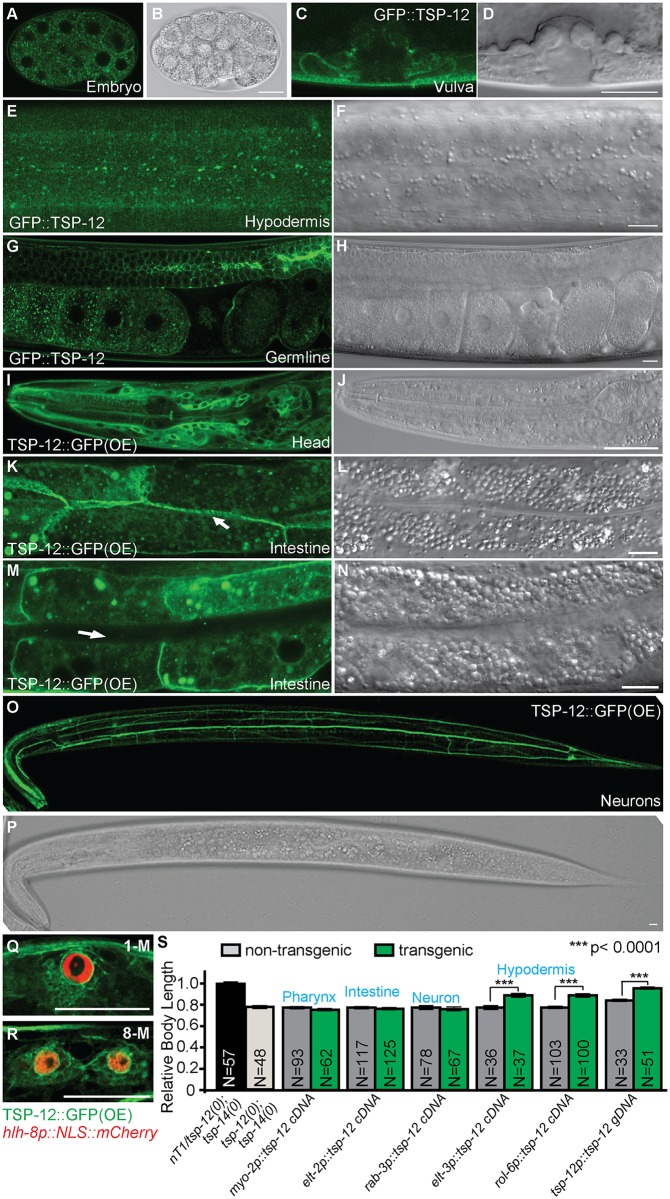
TSP-12 is localized both to the cell surface and in cytoplasmic vesicles in multiple cell types, including known Sma/Mab signal-receiving cells. (A-P) Confocal fluorescent images (A, C, E, G, I, K, M, O) and corresponding DIC images (B, D, F, H, J, L, N, P) of transgenic worms expressing endogenous GFP::TSP-12 (A, C, E, G) or over-expressed (OE) TSP-12::GFP (I, K, M, O) in the early embryo (A), L4 developing vulva (C), late L3 hypodermis (E), young adult gonad (G), L3 larva head (I), L4 intestine (K: basolateral focal plane of the intestine and the arrow points to the lateral membrane, M: middle focal plane of the intestine and the arrow points to the apical lumen of the intestine), and L3 neurons (O). (Q and R) L1 transgenic animals carrying both over-expressed TSP-12::GFP and the M lineage specific reporter *hlh-8p*::*NLS*::*mCherry*. TSP-12::GFP is present in M lineage cells from 1-M (Q) to 8-M (R) stages. Some M lineage cells are out of the focal planes shown. Scale bars represent 10μm in A-R. (S) Rescue of the small body size of *tsp-12(ok239); tsp-14(jj95)* worms produced by *tsp-12(ok239)/+; tsp-14(jj95)* mothers using tissue-specific *tsp-12* cDNA expression. The mean body length of WT worms is normalized to 1.0. Error bars represent 95% confidence intervals for the normalized body length.

To determine how TSP-12 functions to promote BMP signaling, we determined the expression and subcellular localization patterns of TSP-12. We tagged the endogenous TSP-12 with GFP at either the N-terminus or at the C-terminus using CRISPR/Cas9-triggered homologous recombination ([21, 22]; see Materials and Methods). Both tagged proteins are fully functional because when introduced into the *tsp-14(0)* background, neither GFP::TSP-12 nor TSP-12::GFP caused any defects observed for *tsp-12(0); tsp-14(0)* double mutants as described above. Additionally, both GFP::TSP-12 and TSP-12::GFP exhibited the same expression and localization pattern. As shown in Fig 2, GFP::TSP-12 is widely expressed from early embryos through larval development to adults in multiple cell types, which include the vulva precursor cells, the hypodermis, and the germline (Fig 2A to 2H). Within a cell, GFP::TSP-12 is both localized to the cell surface and in the cytoplasm in what appears to be small vesicles (Fig 2A, 2E and 2G). Because the fluorescence signal for the endogenously tagged GFP::TSP-12 in somatic cells is faint, we also generated integrated transgenic lines over-expressing a functional, C-terminally tagged TSP-12::GFP transgene (see Materials and Methods). In these animals, we detected TSP-12::GFP in the pharynx, intestine, neurons and M lineage cells (Fig 2I to 2R). The cell surface TSP-12::GFP in the intestinal cells appears to be restricted to the basolateral membrane but absent in the apical membrane (Fig 2K and 2M).

### TSP-12 functions in the signal-receiving cells to promote BMP signaling in regulating body size and mesoderm development

It has been well established that the Sma/Mab pathway receptors and downstream Smad proteins function in the hypodermal and M lineage cells to regulate body size and mesoderm development, respectively [19, 23, 24]. Since *tsp-12* is expressed in the hypodermal and M lineage cells, we asked whether it functions in these cells to promote Sma/Mab signaling. We forced the expression of *tsp-12* cDNA in specific tissues of the *tsp-12(0); tsp-14(0)* double mutants using tissue specific promoters. Forced expression of the *tsp-12* cDNA in the hypodermis (*elt-3p*::*tsp-12* cDNA and *rol-6p*::*tsp-12* cDNA) rescued the small body size of *tsp-12(0); tsp-14(0)* double mutants, but forced expression in the pharynx (*myo-2p*::*tsp-12* cDNA), intestine (*elt-2p*::*tsp-12* cDNA) or neurons (*rab-3p*::*tsp-12 cDNA*) had no effect (Fig 2S). Forced expression of *tsp-12* in the M lineage (*hlh-8p*::*tsp-12* cDNA) partially rescued the Susm phenotype of *tsp-12(0); tsp-14(0)* double mutants: transgenic *tsp-12(0); tsp-14(0)* mutants carrying the *hlh-8p*::*tsp-12* cDNA transgene exhibited 15% (n = 108) of the Susm phenotype, while non-transgenic *tsp-12(0); tsp-14(0)* mutants exhibited 59% (n = 230) of the Susm phenotype. Thus, TSP-12 functions in the signal-receiving cells to promote Sma/Mab signaling in regulating body size and M lineage development.

### The *C*. *elegans* ADAM10 ortholog SUP-17 functions in the BMP signaling pathway

**Fig 3:**
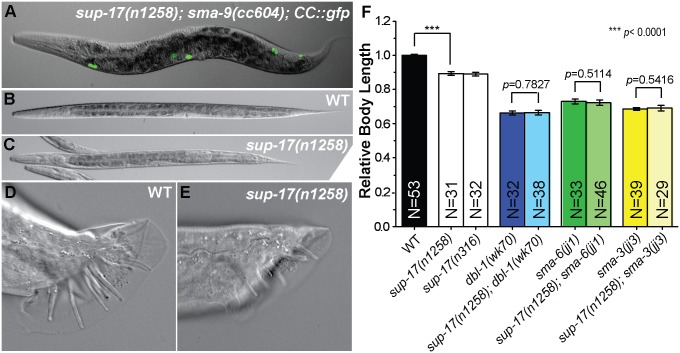
*sup-17* functions in Sma/Mab signaling. (A) Merged GFP and DIC image of a *sup-17(n1258); sma-9(cc604)* animal carrying the *CC*::*gfp* reporter. (B-E) Representative DIC images of developmental stage-matched WT (B, D) and *sup-17(n1258)* (C, E) worms, showing the smaller body size (C) and abnormal male tail (E) of *sup-17(n1258)* worms. (F) Relative body length of stage-matched WT and various mutant worms measured at the L4 stage. Worms have developed to the L4 stage at 25°C from synchronized L1 stage. The mean body length of WT worms is normalized to 1.0. Error bars represent 95% confidence intervals (CI) for the normalized body length.

TSP-12 and TSP-14 orthologs are known to regulate the maturation and trafficking of ADAM10 (a disintegrin and metalloprotease 10) to promote Notch signaling in Drosophila and mammalian cells [13]. We therefore tested whether the *C*. *elegans* ADAM10 ortholog, SUP-17 [25], and its close homolog ADM-4 [26], play any role in Sma/Mab signaling using the *sma-9* M lineage suppression assay. A deletion allele of *adm-4*, *ok265*, failed to suppress the *sma-9* M lineage defect; instead, two strong loss-of-function alleles of *sup-17*, *n1258* and *n316*, both partially suppressed the *sma-9* M lineage defect (Fig 3A and Table 2A; *sup-17* null mutants are embryonic lethal [25] and cannot be used in this assay). Both *sup-17(n1258)* and *sup-17(n316)* mutants also exhibit smaller body sizes (Fig 3B, 3C and 3F). *sup-17(n1258)* males exhibit severe tail patterning defects, including shortened and fused rays, smaller fans (Fig 3D and 3E) and crumpled spicules. Thus, *sup-17* strong loss-of-function mutants exhibit Sma/Mab signaling defects.

**Table 2:** Suppression of the *sma-9* M lineage phenotype by *sup-17* and *unc-40* mutations.

**A. Susm**^a^ **phenotype of *sup-17* mutations.**	
**Genotype**	**% Susm (N)**^b^
*sma-9(cc604)*	—
*adm-4(ok265) sma-9(cc604)*	0% (N = 387)
*sup-17(n316); sma-9(cc604)*	31.4% (N = 379)
*sup-17(n1258); sma-9(cc604)* [20°C]	78.6% (N = 751)
*sup-17(n1258); sma-9(cc604)* [25°C]	86.1% (N = 607)
*sup-17(n1258); sma-9(cc604); jjEx[sup-17p*::*sup-17 genomic*::*gfp*]^d^	40.1% (N = 379) *** ^c^
*sup-17(n1258); sma-9(cc604); jjEx[sup-17p*::*sup-17 cDNA*]^e^	40.9% (N = 653) *** ^c^
*sup-17(n1258); sma-9(cc604); jjEx[hlh-8p*::*sup-17 cDNA*]^f^	63.6% (N = 176) * ^c^
*sup-17(n1258); sma-9(cc604); jjEx[rab-3p*::*sup-17 cDNA*]^g^	75.7% (N = 136) ^ND^ ^c^
**B. Susm**^a^ **phenotype of *sel-12* and *hop-1* mutations.**	
**Genotype**	**% Susm (N)**
*sel-12(ok2078) sma-9(cc604)*	0% (N = 100)
*sel-12(ar171) sma-9(cc604)*	0% (N = 100)
*hop-1(ar179); sma-9(cc604)*	0.3% (N = 326)
*hT2[qIs48]/hop-1(ar179); sel-12(ok2078) sma-9(cc604)*	6.9% (N = 261)
*hop-1(ar179); sel-12(ok2078) sma-9(cc604)*	3.3% (N = 92)
**C. Susm**^a^ **phenotype of *sup-17* and *unc-40* mutations.**	
**Genotype**	**% Susm (N)**
*sup-17(n1258); sma-9(cc604)*	71.1% (N = 622)
*unc-40(e1430); sma-9(cc604)*	94.2% (N = 345)
*unc-40(e1430) sup-17(n1258); sma-9(cc604)*	95.4% (N = 382)
*unc-40(ev495); sma-9(cc604)*	0% (N = 130)
*unc-40(ev495) sup-17(n1258); sma-9(cc604)*	46.2% (N = 195)
*unc-40(tr115); sma-9(cc604)*	0.9% (N = 109)
*unc-40(tr115) sup-17(n1258); sma-9(cc604)*	47.6% (N = 341)

In the absence of the *sma-9(cc604)* mutation, none of the single or double mutants listed above has any defects affecting the number of M-derived CCs.

^a^ Unless noted, the Susm phenotype of various mutants was examined at 20°C.

^b^ The Susm penetrance refers to the percent of animals with 1–2 M-derived CCs as scored by the *CC*::*gfp* reporter. Up to 3% of *sma-9(cc604)* single mutants have 1–2 M-derived CCs, as reported in Foehr et al. [19].

^**c**^ Statistical analysis was conducted by comparing the transgenic *vs*. non-transgenic animals using unpaired two-tailed Student’s *t*-test.

***p< 0.0001, * *p*< 0.05, ND: not different.

^**d**^ Data were compiled from analyzing two independent transgenic lines: *jjEx4451[sup-17p*::*sup-17 genomic*::*gfp*] and *jjEx4452[sup-17p*::*sup-17 genomic*::*gfp*].

^**e**^ Data were compiled from analyzing two independent transgenic lines: *jjEx4569[sup-17p*::*sup-17 cDNA*] and *jjEx4572[sup-17p*::*sup-17 cDNA*].

^**f**^ Data were compiled from analyzing two independent transgenic lines: *jjEx3469[hlh-8p*::*sup-17 cDNA*] and *jjEx4315[hlh-8p*::*sup-17 cDNA*].

^**g**^ Data were compiled from analyzing two independent transgenic lines: *jjEx4410[rab-3p*::*sup-17 cDNA*] and *jjEx4411[rab-3p*::*sup-17 cDNA*].

We then performed double mutant analysis between *sup-17(n1258)* and null mutations in the Sma/Mab pathway by assaying for their body sizes. We found that *sup-17(n1258); dbl-1(wk70)*, *sup-17(n1258); sma-6(jj1)* and *sup-17(n1258); sma-3(jj3)* double mutants were as small as the corresponding *dbl-1(wk70)*, *sma-6(jj1)* or *sma-3(jj3)* single mutants, respectively (Fig 3F). This lack of enhancement of the small body size phenotype of Sma/Mab pathway mutants by *sup-17(n1258)* is not because Sma/Mab pathway mutants have reached the minimal body size of *C*. *elegans*, because previous studies have shown that mutations in genes regulating body size but not functioning in the Sma/Mab pathway, such as *sma-5* (encoding a MAPK, [27] or *wts-1* (encoding a serine/threonine kinase, [28]), can make Sma/Mab pathway mutants much smaller. We therefore concluded that SUP-17 functions in the Sma/Mab pathway to regulate body size.

### SUP-17 is expressed and functions in the signal-receiving cells to promote BMP signaling in in regulating body size and mesoderm development

**Fig 4:**
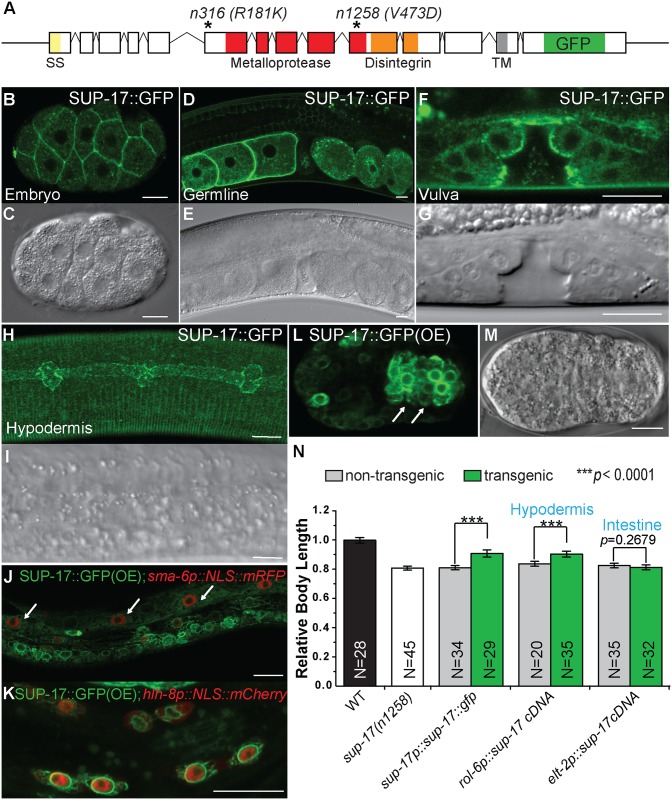
SUP-17 is localized both to the cell surface and in the cytoplasm in multiple cell types, including known Sma/Mab signal-receiving cells. (A) A schematic of the *sup-17* genomic region (not to scale), depicting the location of various protein domains, the GFP insertion site and the location of the *n1258* and *n316* molecular lesions. (B-I) Confocal fluorescent images (B, D, F, H) and corresponding DIC images (C, E, G, I) of transgenic worms expressing endogenous SUP-17::GFP (B, D, F, H) in the early embryo (B), gravid adult gonad (D), L4 developing vulva (F) and L3 hypodermis (H). (J) An L3 transgenic animal carrying both over-expressed SUP-17::GFP and *sma-6p*::*NLS*::*mRFP*, showing expression of SUP-17::GFP in the hypodermal cells (arrows). (K) An L1 transgenic animal carrying both over-expressed SUP-17::GFP and the M lineage specific reporter *hlh-8p*::*nls*::*mCherry* at the 16-M stage. Some M lineage cells are out of the focal planes shown. (L-M) GFP (L) and DIC (M) images of a mid-stage transgenic embryo carrying over-expressed SUP-17::GFP, showing GFP expression in the intestinal precursor cells (marked by arrows). Anterior is to the left. Scale bars represent 10μm in B-M. (N) Rescue of the small body size of *sup-17(n1258)* worms by tissue-specific *sup-17* cDNA expression. The mean body length of WT worms is normalized to 1.0. Error bars represent 95% confidence intervals for the normalized body length.

To determine how SUP-17 functions in modulating Sma/Mab signaling, we tagged the endogenous SUP-17 by generating SUP-17::GFP knock-in strains using CRISPR/Cas9-triggered homologous recombination (see Materials and Methods). The SUP-17::GFP fusion is functional, because a transgene with the same SUP-17::GFP fusion rescued the small body size and Susm phenotypes of *sup-17(n1258)* mutants (Fig 4L). Like TSP-12, SUP-17::GFP is also widely expressed from the early embryo through larval development to adults. Cells expressing SUP-17::GFP include the germline, developing vulva, hypodermis, M lineage cells and intestinal cells (Fig 4B to 4M). Also like TSP-12, SUP-17::GFP is localized both at the cell surface and in the cytoplasm in small puncta (Fig 4B, 4D, 4F and 4H). In the early embryo, cell surface localized SUP-17::GFP is enriched in the basolateral, but not the apical, surfaces (Fig 4B). Forced expression of *sup-17* cDNA in the hypodermis (*rol-6p*::*sup-17* cDNA), but not in the intestine (*elt-2p*::*sup-17* cDNA), rescued the small body size of *sup-17(n1258)* mutants (Fig 4N). Similarly, forced expression of *sup-17* cDNA in the M lineage (*hlh-8p*::*sup-17* cDNA) partially rescued the Susm phenotype of *sup-17(n1258)* mutants (Table 2A). These findings indicate that SUP-17 also functions in the signal-receiving cells to modulate Sma/Mab signaling in regulating body size and mesoderm development.

### TSP-12 can bind to SUP-17 and promote its cell surface localization

**Fig 5:**
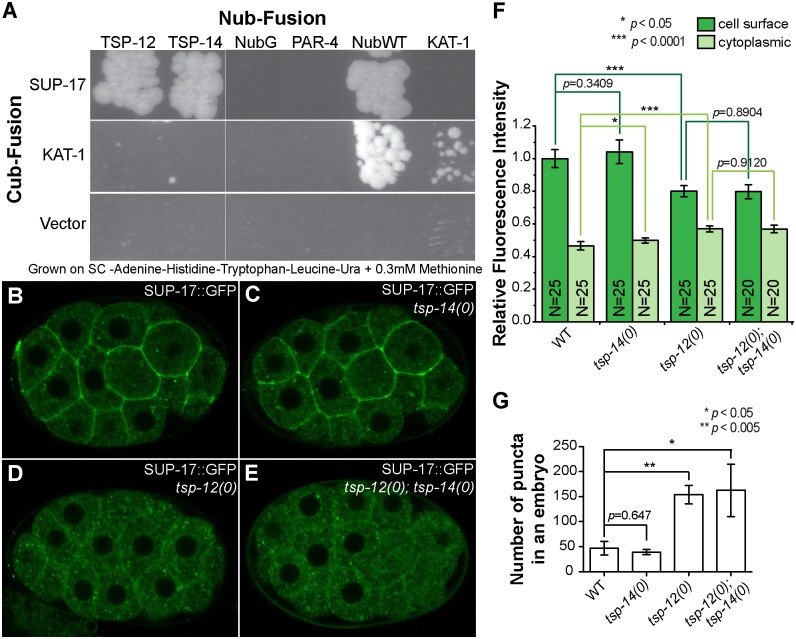
TSP-12 binds to and promotes the cell surface localization of SUP-17. (A) Mating-based split ubiquitin yeast-two-hybrid assay showing that SUP-17 can bind to TSP-12 and TSP-14. (B-E) Confocal images showing the localization of endogenously-tagged SUP-17::GFP in WT (B), *tsp-14(0)* (C), *tsp-12(0)* (D) and *tsp-12(0); tsp-14(0)* (E) backgrounds. (F) Quantification of relative fluorescence intensity of cell surface and cytoplasmic SUP-17::GFP in WT and various mutants (see Materials and Methods). A total of 20 or 25 cells, five cells/30-cell stage embryo, was measured. The mean cell surface fluorescence intensity in WT embryos is normalized to 1.0. Error bars represent 95% confidence intervals (CI) for the normalized mean fluorescence intensity. (G) Quantification of the number of intra-cellular puncta in embryos of WT and various mutants (see Materials and Methods). Three to five 30-cell stage embryos were measured. Error bars represent standard error of the mean (SEM).

The shared expression/localization pattern and function of TSP-12, TSP-14 and SUP-17 in Sma/Mab signaling suggest that TSP-12 and TSP-14 may be functionally linked with SUP-17 in their roles in the Sma/Mab pathway. Using the mating-based split ubiquitin yeast-two-hybrid assay for membrane protein interactions (see Materials and Methods), we found that SUP-17 can bind to TSP-12 and TSP-14 (Fig 5A). We then examined the localization of SUP-17::GFP in *tsp-12(0)* and *tsp-14(0)* single mutants, and in *tsp-12(0); tsp-14(0)* double mutants. We focused on the early embryo for these localization studies because of the large cell sizes and the brighter GFP signals in these embryos. Wild-type embryos expressing the endogenously-tagged SUP-17::GFP showed GFP localization both at the cell surface and in the cytoplasm (Fig 5B and 5F). This localization pattern does not change in *tsp-14(0)* mutant embryos (Fig 5C and 5F). However, in *tsp-12(0)* or *tsp-12(0); tsp-14(0)* mutant embryos, there is a significant reduction of cell surface SUP-17::GFP accompanied by an increase of intracellular SUP-17::GFP (Fig 5D, 5E and 5F) and an increase in the number of bright puncta in the cytoplasm (Fig 5G). These findings indicate that TSP-12 and TSP-14 can bind to SUP-17 and that TSP-12, but not TSP-14, promotes the cell surface localization of SUP-17 in embryos.

### The two *C*. *elegans* presenilins SEL-12 and HOP-1 do not appear to be required for Sma/Mab signaling

The involvement of SUP-17/ADAM10 in Sma/Mab signaling prompted us to test whether the γ-secretase functions in Sma/Mab signaling, because Notch undergoes sequential cleavage by ADAM and γ-secretase [29]. *C*. *elegans* has three paralogous genes encoding presenilins, *sel-12*, *hop-1* and *spe-4* [30–32], and presenilins are major components of the γ-secretase complex. Because *spe-4* appears to be specifically expressed and functions in the germline during spermatogenesis, and because *sel-12* and *hop-1* share redundant functions, in particular in Notch signaling [30–32], we tested whether mutations in *sel-12* and *hop-1* exhibited any Susm phenotypes. Using null alleles for each gene, we found that *sel-12(ar171)*, *sel-12(ok2078)*, *hop-1(ar179)* single mutants or *hop-1(ar179); sel-12(ar171)* and *hop-1(ar179); sel-12(ok2087)* double mutants did not exhibit any Susm phenotype (Table 2B), suggesting that the γ-secretase is unlikely to be involved in Sma/Mab signaling in *C*. *elegans*.

### The neogenin homolog UNC-40 is one of the substrates of SUP-17/ADAM10 in Sma/Mab signaling

ADAM10 is known to cleave transmembrane or membrane-associated proteins in a process called ectodomain shedding [33, 34]. We have previously shown that the extracellular domain of UNC-40 is sufficient to function in promoting Sma/Mab signaling, suggesting that UNC-40 may be cleaved for its function in Sma/Mab signaling [18]. We therefore tested genetically whether UNC-40 is a SUP-17/ADAM10 substrate. We reasoned that if UNC-40 is a SUP-17 substrate in BMP signaling, then the extracellular domain of UNC-40, which is sufficient to function in promoting Sma/Mab signaling, should be able to, either completely or partially, rescue the Sma/Mab signaling defects of *sup-17* mutants. We, therefore, tested whether two *unc-40* alleles that lead to the production of truncated UNC-40 proteins lacking the intracellular domain (ICD), *ev495* and *tr115* [18, 35], could rescue the Susm phenotype of *sup-17(n1258)* mutants. As shown in Table 2C, a null *unc-40* allele, *e1430*, did not rescue the Susm phenotype of *sup-17(n1258)* mutants, while both *unc-40(ev495)* and *unc-40(tr115)* partially suppressed the Susm phenotype of *sup-17(n1258)* mutants. The similar penetrance of the Susm phenotype shared by *unc-40(e1430)* and *unc-40(e1430) sup-17(n1258)* mutants, and the partial suppression of the *sup-17(n1258)* Susm phenotype by *unc-40(ev495)* and *unc-40(tr115)* suggest that: 1) UNC-40 and SUP-17 function in the same pathway to regulate Sma/Mab signaling, and 2) UNC-40 is a substrate of SUP-17 in the Sma/Mab pathway. The incomplete suppression of the Susm phenotype of *sup-17(n1258)* mutants by *unc-40(ev495* or *tr115)* suggests that additional SUP-17 substrates must exist in the Sma/Mab pathway.

## Discussion

### The TspanC8 tetraspanins and ADAM10 positively promote BMP signaling independent of their roles in Notch signaling

In this study, we extended our previous work [7] and provided additional evidence that TSP-12 and TSP-14, two paralogous TspanC8 tetraspanins, function redundantly to promote BMP signaling in *C*. *elegans*. We further showed the ADAM10 ortholog SUP-17 also functions in the BMP pathway. Both *tsp-12; tsp-14* double mutants and *sup-17* single mutants exhibited BMP signaling defects, including a smaller body size, male tail abnormalities, reduced RAD-SMAD reporter activity, and suppression of the *sma-9* mesoderm phenotype (Susm). Both TSP-12 and SUP-17 are expressed and function in the signaling-receiving cells to promote BMP signaling in regulating body size and M lineage development. TSP-12 and TSP-14 can bind to SUP-17. TSP-12 and SUP-17 share similar expression and subcellular localization patterns. Additionally, TSP-12 promotes the cell surface localization of SUP-17 in embryos.

TspanC8 tetraspanins and ADAM10 are known to promote Notch signaling in *C*. *elegans*, *Drosophila*, and mammalian tissue culture cells [12, 15, 25, 36, 37]. Three lines of evidence suggest that the function of these proteins in promoting BMP signaling is independent of their role in Notch signaling. First, unlike *lin-12(0)* mutants, which exhibit a ventral-to-dorsal fate transformation in the M lineage [38], *tsp-12(0)*, *tsp-14(0)*, *tsp-12(0); tsp-14(0)* or *sup-17(n1258)* mutants do not exhibit any M lineage defects. Second, two *lin-12* null mutants, *lin-12(n941)* and *lin-12(ok2215)*, do not exhibit any body size defects (S1 Table). Third, we have previously reported that *lin-12(0)* mutants do not suppress the M lineage defects of *sma-9(0)* mutants [39]. Thus our findings uncover an additional function for TspanC8 tetraspanins and ADAM10 in promoting BMP signaling. Notably, TSP-12 and TSP-14 may have other functions in addition to modulating Notch and BMP signaling. In particular, *tsp-12(0); tsp-14(0)* double mutant embryos exhibited ventral enclosure defects, but Notch and BMP signaling pathways have not been previously implicated in the regulation of ventral enclosure in *C*. *elegans* [40].

Over-expression and knockdown experiments in *Drosophila* and mammalian tissue culture cells have shown that TspanC8 tetraspanins promote the cell surface localization of ADAM10 [12–14]. Our work demonstrates that this function of the TspanC8 tetraspanins is evolutionarily conserved. How TspanC8 tetraspanins regulate the cell surface localization of ADAM10 requires further investigation. Tetraspanins are known to regulate intracellular trafficking of various cargo proteins via multiple distinct mechanisms [11, 41]. Previous work showed that TspanC8 tetraspanins function to promote the exit of ADAM10 from the endoplasmic reticulum (ER) [12–14]. We noted in our work that SUP-17::GFP from transgenic lines overexpressing this transgene exhibited a strong perinuclear, ER-like localization pattern with very little cell surface localization (Fig 4J and 4K). However, endogenously-tagged SUP-17::GFP exhibited much more prominent cell surface localization (Figs 4B, 4D, 4F, 4H and 5B). We showed that this cell surface localization is significantly reduced in *tsp-12(0)* and *tsp-12(0); tsp-14(0)* mutants; while there was a concomitant increase of bright SUP-17::GFP-positive puncta in these mutant embryos (Fig 5D to 5G). Whether these signals represent SUP-17::GFP trapped in the ER or in other intracellular organelles will need further investigation.

The basolateral plasma membrane localization of TSP-12 in intestinal cells (Fig 2K and 2N) resembles the localization patterns of both SMA-6/RI and DAF-4/RII of the BMP pathway [6]. We have previously shown that both TSP-12 and TSP-14 can bind to SMA-6 in yeast [7]. Whether TSP-12, TSP-14 or both of them are required for the proper localization of SMA-6 and DAF-4 will require further investigation.

Our phenotypic analysis clearly showed that TSP-12 and TSP-14 function redundantly to promote BMP signaling (Fig 1F and 1K). Both proteins can also bind to SUP-17 in yeast (Fig 5A). However, only *tsp-12(0)* mutant embryos showed severe defect in SUP-17::GFP localization, and this defect is not enhanced in *tsp-12(0); tsp-14(0)* double mutant embryos (Fig 5D to 5G). Due to technical challenges, our SUP-17::GFP localization study was carried out in embryos but not in tissues relevant to Sma/Mab signaling. It is possible that TSP-14 may function similarly as TSP-12 to promote the cell surface localization of SUP-17 in other cell types, but not in the early embryo. Alternatively, it is possible that while TSP-12 and TSP-14 exhibit functional redundancy in regulating BMP signaling and other developmental processes at the organismal level, they may differ in their functions at the sub-cellular and/or molecular level.

### The role of SUP-17/ADAM10 in the BMP signaling pathway

In worms, flies and mammals, ADAM proteases are well known for their role in processing the Notch receptor upon ligand binding [42–44]. Notch undergoes sequential cleavage, first by the ADAM protease, followed by subsequent cleavage by the γ-secretase. Although ADAM10 has not been previously implicated in BMP signaling in any organisms, the ADAM10 paralog ADAM17 or TACE (tumor necrosis factor-alpha-converting enzyme) has been shown to play a role in TGFβ signaling in mammals [45]. Earlier work by Liu and colleagues indicated that ADAM17/TACE-mediated cleavage of the type I TGFβ receptor (TβR1) causes downregulation of TGFβ signaling in CHO cells [45]. Cleavage of TβR1 by ADAM17/TACE and Presenilin 1, a γ-secretase catalytic core component, has also been found in multiple cancer cell lines, which results in the translocation of the intracellular domain (ICD) of TβR1 into the nucleus and activation of genes involved in tumor invasion [46, 47]. More recently, polymorphic variants of the *Adam17* gene have been found to differentially regulate TGFβ signaling and influence the severity of *Tgfb1*-dependent vascular pathology in mice and humans [48]. Because ADAM10 and ADAM17 are known to share a number of common substrates [34], and in *C*. *elegans* SUP-17/ADAM10 and ADM-4/ADAM17 share redundant functions [26], our findings raise the possibility that ADAM10 or ADAM17 may also play a role in regulating BMP signaling in mammals. Consistent with this notion, Jackson and colleagues reported that ADAM17/TACE-null mice exhibit defects in valvulogenesis that are associated with aberrant BMP signaling [49].

How SUP-17/ADAM10 functions to modulate BMP signaling is an open question. Our genetic studies suggest that the neogenin/DCC ortholog UNC-40 is one of the substrates of SUP-17. UNC-40, like its vertebrate ortholog neogenin, functions through the RGM (repulsive guidance molecule) proteins to promote BMP signaling [10, 18]. We have previously shown that the extracellular domains of UNC-40 and neogenin are sufficient to mediate BMP signaling in *C*. *elegans* and in mammalian tissue culture cells, respectively [18]. There are other reports showing that neogenin or DCC can be cleaved by ADAM proteases. Earlier work using ADAM inhibitors and rat dorsal spinal cord explants showed that DCC undergoes ADAM-dependent proteolytic processing, which may affect its role in regulating axon migration [50]. Okamura and colleagues showed that ADAM17/TACE can bind to and cleave neogenin, regulating the behavior of rat cortical neurons in response to RGM [51]. Recently, van Erp and colleagues showed that ADAM17/TACE-mediated cleavage of neogenin is regulated by the leucine-rich repeats and immunoglobulin-like domains (Lrig) protein Lrig2, and that this regulation is required for RGM-mediated neurite growth inhibition in vitro and cortical neuron migration in vivo [52]. How SUP-17 cleaves UNC-40 and whether similar types of regulatory interactions also occur in the BMP signaling pathway will require further experimentation. Nevertheless, cleavage of UNC-40 by SUP-17 may not be followed by subsequent cleavage by the γ-secretase, because *hop-1(0); sel-12(0)* double mutants, which lack two of the redundant *C*. *elegans* presenilins, do not exhibit any BMP signaling defects (Table 2). We are aware that we assayed the Susm phenotype in *hop-1(0); sel-12(0)* animals produced by *hop-1(0)/+; sel-12(0)* mothers due to the sterility of the *hop-1(0); sel-12(0)* double mutants. Thus, although unlikely, we cannot rule out the possibility that residual γ-secretase activity is still present in *hop-1(0); sel-12(0)* double mutants due to maternally loaded HOP-1 and that this residual activity is sufficient for proper Sma/Mab signaling.

Our genetic studies suggested that UNC-40/neogenin is not the only substrate of SUP-17/ADAM10 in the BMP pathway. Identifying the additional substrate(s) and determining the functional consequences of ADAM processing of these substrates in vivo will provide insight into the intricate levels of modulation of BMP signaling. Notably, ADAM10 and ADAM17 mutations are associated with many diseases in humans, including a variety of cancers [34, 53, 54]. We speculate that some of these incidences could be due to abnormal processing of factors involved in BMP signaling caused by altered activities of ADAM10 and/or ADAM17.

## Materials and Methods

### *C*. *elegans* strains

All strains were maintained at 20°C using standard culture conditions [55] unless otherwise noted. The following mutations, transgenes and balancers were used: Linkage group I (LG I): *drag-1(jj4)*, *hop-1(ar179)*, *unc-40(n1430)*, *unc-40(ev495)*, *unc-40(tr115)*, *sup-17(n316)*, *sup-17(n1258)*, *sup-17*::*gfp(jj98)*, *arIs37(secreted CC*::*gfp)*; LG II: *sma-6(jj1)*; *jjIs2437 (RAD-SMAD)*; LG III: *lon-1(jj67)*, *sma-3(jj3)*, *lin-12(n941)*, *lin-12(ok2215)*, *ccIs4438(intrinsic CC*::*gfp)*, *hT2[qIs48]*; LG IV: *tsp-12(ok239)*, *gfp*::*3*×*flag*::*tsp-12(jj181)*, *gfp*::*3*×*flag*::*tsp-12(jj182); nT1[qIs51]*; LG V: *dbl-1(wk70)*, *him-5(e1467)*; LG X: *sel-12(ar171)*, *sel-12(ok2078)*, *lon-2(e678)*, *tsp-14(jj95)*, *tsp-14(jj96)*, *tsp-14(jj97)*, *adm-4(ok265)*, *sma-9(cc604)*.

### Body size measurement and RAD-SMAD reporter assay

Body size measurement and RAD-SMAD reporter assays were performed following the protocols described in Tian et al. [18]. Hermaphrodite worms at the L4 stage (identified based on vulva development) were used for body size measurement. Body sizes were measured using segmented lines in Fiji [56] after images were taken. Synchronized L3 hermaphrodites carrying the RAD-SMAD reporter *jjis2437* II [18] were used for imaging. The reporter fluorescence intensity was measured using Fiji.

### Oligonucleotides, plasmid constructs and transgenic lines

Oligonucleotides used in this study are listed in SI Appendix, S2 Table. Plasmids generated are listed in SI Appendix, S3 Table. The *tsp-12* cDNA was amplified from the Vidal RNAi library [57], while *yk23h2* contains the full-length *sup-17* cDNA. Each clone contains a point mutation in the respective coding regions of *tsp-12* and *sup-17*, which were respectively fixed via site-directed mutagenesis, resulting in the generation of pJKL1079 (containing the *tsp-12* ORF), and pJKL1140 (containing the *sup-17* cDNA). pK3, a rescuing plasmid containing 7kb of the *sup-17* genomic region, is a kind gift from Iva Greenwald (Columbia University).

One of the following plasmids were used as co-injection markers to generate transgenic lines: pRF4 (*rol-6(su1006)*), LiuFD61 (*mec-7p*::*mRFP*), pJKL449 (*myo-2p*::*gfp*::*unc-54 3’UTR*), or pCFJ90 (*myo-2p*::*mCherry*::*unc-54 3*’*UTR)* [58]. Stable integrants carrying pZL51 were generated using γ-radiation, followed by outcrossing with N2 animals five times.

### Generating *tsp-14* deletion alleles using the dual sgRNA-guided CRISPR/Cas9 system

To generate deletion alleles of *tsp-14*, we designed two sgRNAs targeting both the N-terminus (ACCACCGCGCGAGCTCGCGT) and the C-terminus (AAGCGGCTGAAGGCATCCG) of the *tsp-14* coding region (SI Appendix, S1 Fig). The sgRNA plasmids pZL8 and pZL9 (S3 Table) were generated by replacing the *unc-119* gRNA sequence in the *pU6*::*unc-119* sgRNA vector [59] with the *tsp-14* sgRNA sequence. The following plasmid mix was injected into young hermaphrodite N2 adults: (1) the Cas9 expression plasmid pDD162 (*peft-3-cas9+empty sgRNA*) [60], (2) two *tsp-14*-specific sgRNA plasmids pZL8 and pZL9, and (3) the co-injection marker pRF4 (*rol-6d*). Positive Rol F1 worms were picked and placed onto fresh NGM plates (3 worms/plate). After they have laid eggs, the F1 worms were pooled for PCR screening using primers ZL4 and ZL5 (S2 Table). After screening 282 F1s, we obtained three *tsp-14* deletion alleles, *jj95*, *jj96* and *jj97*, which were confirmed by Sanger sequencing (SI Appendix, S1 Fig).

### Generating endogenously tagged TSP-12::GFP or SUP-17::GFP strains using CRISPR/Cas9-mediated homologous recombination

We tagged the endogenous TSP-12 at either the N-terminus or the C-terminus using CRISPR/Cas9. To generate these TSP-12 knock-ins, we followed the strategy described in Arribere et al. [21] to generate two sgRNA expression plasmids for each tagging strategy (see S2 and S3 Tables for oligo and plasmid information). We then used the method described in Dickinson et al. [22] to generate the homologous repair templates, and to subsequently obtain stable knock-in strains that carry either GFP::3×FLAG::TSP-12 (*jj181* and *jj182*) or TSP-12::GFP::3×FLAG (*jj194* and *jj196*). We first confirmed the success of knock-ins by genotyping, and then tested *jj181* and *jj194* for functionality by crossing each of them into the *tsp-14(0)* background and examined the strains for viability and fertility.

The *sup-17*::*gfp* knock-in strain was generated by injecting the following mix of plasmid DNAs into N2 worms: (1) a Cas9 expression plasmid pDD162 [60], (2) two sgRNA plasmids (pLW4 and pLW5, generated using the *pU6*::*unc-119* sgRNA plasmid described in Friedland et al. [59]), (3) the homologous repair template pJKL1034 (SUP-17::GFP translational fusion, with GFP inserted between amino acid 876 and 877, see S3 Table), and (4) a co-injection marker pRF4 (*rol-6d*). GFP knock-in events were screened via PCR using primers LW27, LW30 and JKL269 (see S2 Table). Single worm PCR of 33 F1 rollers failed to detect any germline integration event. However, we screened the F3 worms from six high transmission efficiency (>30%) *rol* transgenic lines and found that five of the six lines examined had a *gfp* knock-in event. After genotyping and confirming homozygous *sup-17*::*gfp* knock-ins, we kept three of the lines, *jj98*, *jj99* and *jj100*, for further analysis.

### Microscopy

To examine the male tail, the developing vulva or developing embryos, animals were visualized under a Leica DMRA2 compound microscope equipped with a Hamamatsu Orca-ER camera using the iVision software (Biovision technology, Inc.). An inverted Zeiss LSM880 confocal microscope with Airyscan was used to examine the expression and subcellular localization patterns of GFP::3×FLAG::TSP-12 or SUP-17::GFP. Fluorescence was detected with an Airyscan detector using the 40× objective (NA 1.4, WD 0.13 mm) and in the super-resolution mode (pinhole is around 2.25 AU). The laser line for GFP excitation is at 488 nm, and for mCherry is at 561 nm. Collected images were subsequently processed by ZEN (Carl Zeiss), and further quantification of the fluorescence signals was performed using Fiji.

To quantify the fluorescence intensity and bright intracellular puncta of SUP-17::GFP, live embryos from worms of different genotypes that were grown under the same condition were collected on the same day and imaged using the same settings as described above. Images of stage-matched embryos were further processed in the same way using Fiji. Average SUP-17::GFP fluorescence intensity was calculated by drawing a straight line of fixed length (for cell surface signal) or by drawing an oval of fixed width and height (for intracellular signal). Only cells with focal plane going through the nucleus were used for this purpose. Bright puncta were defined as those signals that are above a threshold of brightness (3,500 pixels) and size (the area is above 0.07 μm^2^). Signals on top of the cell membrane were excluded from the counting.

### Split ubiquitin yeast two-hybrid assay

The split-ubiquitin yeast two-hybrid experiments were carried out by following the protocol described in Grefen et al. [61]. The bait CubPLV and prey NubG fusion constructs used are listed in SI Appendix, S2 Table. After transforming into yeast, interactions among each pair of bait and prey constructs were visualized by streaking diploid cells on SC-Trp, -Leu, -Ade, -His, -Ura, -Met plates that were supplemented with four different concentrations of methionine: 0mM, 0.075mM, 0.150mM, 0.300mM, respectively. Growth was monitored for 2–9 days at 30°C. The plasmids KAT-1-Cub-PLV, KAT-1-NubG (in XN21 vector), empty NubG vector, and the vector expressing soluble wild-type Nub (NubWT) were used as controls [7]. Confirmation of expression of each fusion protein was by western blot analysis using rabbit polyclonal anti-VP16 antibodies (ab4808, Abcam, for CubPLV fusions) and monoclonal anti-HA antibodies (Clone 12CA5, Sigma, for NubG fusions).

### Statistical analyses

Statistical analyses were conducted with Prism 5 (GraphPad), and the test for significance for groups of two were analyzed using the unpaired Student’s t-test. Two-tailed *p* values were calculated. Data was shown as mean ± 95% confidence interval (CI).

## Supporting Information

S1 Fig Diagram of the *tsp-14* genomic region and the knockout alleles generated by the dual sgRNA-guided CRISPR/Cas9 system. Two target sgRNA sequences (in red) were designed to delete about 2.9kb of the *tsp-14* genomic region, which will delete both *tsp-14* isoforms a and b. The genomic sequences around the deleted region are shown.
(TIF)

S1 Movie The DIC movie showing embryonic development of a *tsp-12(ok239)* embryo. Embryogenesis lasted about 13 hours at room temperature and the image sequences were captured one frame per minute. The movie has 769 frames.
(AVI)

S2 Movie The DIC movie showing embryonic development of two *tsp-12(ok239); tsp-14(jj95)* embryos produced by homozygous *tsp-12(ok239); tsp-14(jj95)* mother worms. The image sequences were captured one frame per minute. The movie has 800 frames.
(AVI)

S1 Table *lin-12* null mutants do not exhibit defects in body length. (DOCX)

S2 Table Oligonucleotides used in this study. (DOCX)

S3 Table Plasmid constructs generated in this study. (DOCX)
