# Supplementary figures and images for: Two Paralogous Tetraspanins TSP-12 and TSP-14 Function with the ADAM10 Metalloprotease SUP-17 to Promote BMP Signaling in Caenorhabditis elegans
Source: PLoS Genet. 2017 Jan 9;13(1):e1006568. doi: 10.1371/journal.pgen.1006568 (PMC5261805; doi:10.1371/journal.pgen.1006568)

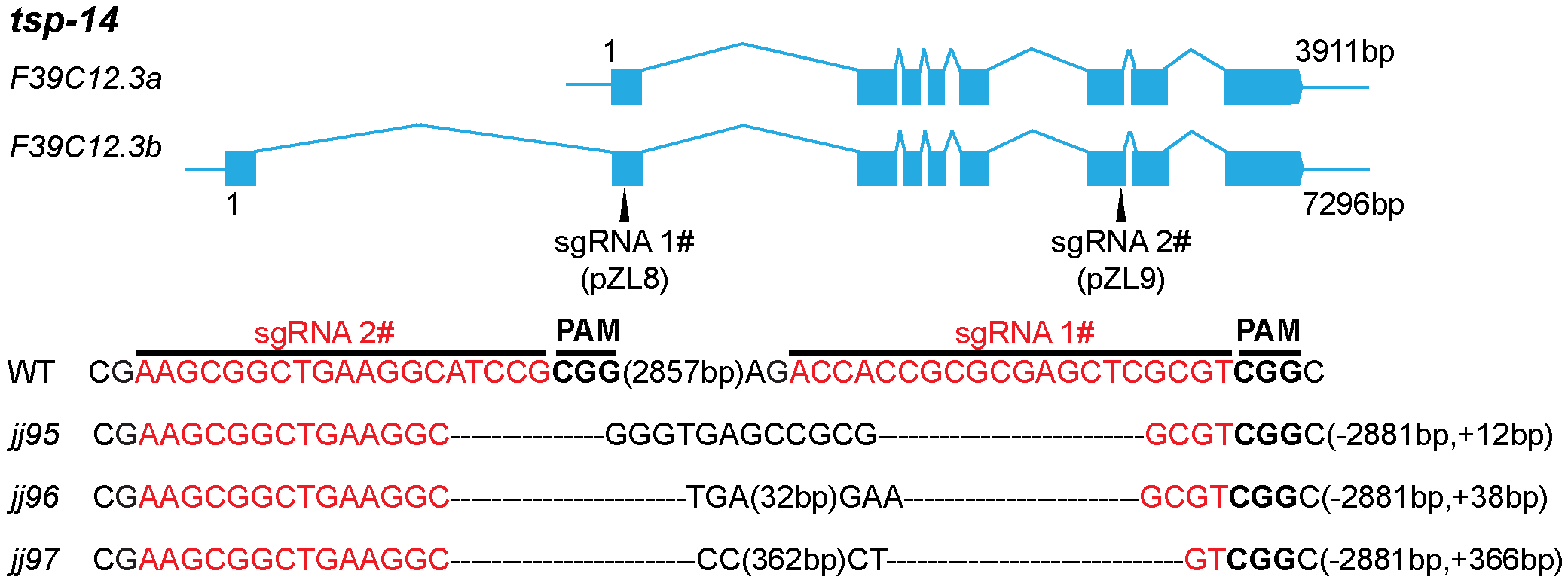

Supplement: S1 Fig — Two target sgRNA sequences (in red) were designed to delete about 2.9kb of the tsp-14 genomic region, which will delete both tsp-14 isoforms a and b. The genomic sequences around the deleted region are shown. (TIF) [file pgen.1006568.s001.tif]
