# Supplementary material for: Two Paralogous Tetraspanins TSP-12 and TSP-14 Function with the ADAM10 Metalloprotease SUP-17 to Promote BMP Signaling in Caenorhabditis elegans
Source: PLoS Genet. 2017 Jan 9;13(1):e1006568. doi: 10.1371/journal.pgen.1006568 (PMC5261805; doi:10.1371/journal.pgen.1006568)
Supplement: S1 Table — (DOCX) [file pgen.1006568.s004.docx]

**Table S1. *lin-12* null mutants do not exhibit defects in body length.**

| **Genotype** | **Relative body length**^a^  **(Mean±95% CI)** | **N** |
| --- | --- | --- |
| WT | 1.00 ± 0.003 | 49 |
| *hT2[qIs48]/lin-12(n941)* isolate 1 | 1.00 ± 0.004 | 107 |
| *lin-12(n941)* isolate 1 | 1.00± 0.006 | 65 |
| *hT2[qIs48]/lin-12(n941)* isolate 2 | 1.00 ± 0.005 | 83 |
| *lin-12(n941)* isolate 2 | 0.99± 0.005 | 73 |
| *hT2[qIs48]/lin-12(ok2215)* | 1.00 ± 0.004 | 74 |
| *lin-12(ok2215)* | 1.00 ± 0.008 | 69 |

^a^ Body length of worms at the L4 Christmas tree stage regarding vulva development were examined. The length of wild-type N2 worms is normalized as 1.00. The body length of *lin-12(0)* worms is not statistically different from that of the control *hT2[qIs48]/lin-12(0)* or wild-type N2 worms.
