# Supplementary material for: Two Paralogous Tetraspanins TSP-12 and TSP-14 Function with the ADAM10 Metalloprotease SUP-17 to Promote BMP Signaling in Caenorhabditis elegans
Source: PLoS Genet. 2017 Jan 9;13(1):e1006568. doi: 10.1371/journal.pgen.1006568 (PMC5261805; doi:10.1371/journal.pgen.1006568)
Supplement: S2 Table — (DOCX) [file pgen.1006568.s005.docx]

**Table S2. Oligonucleotides used in this study.**

| **Oligonucleotides for genotyping mutants** |
| --- |
| *sup-17(n1258)*:  LW23 (AGAGTGTCAACCTGGTCTTC, F)  LW25 (TGAGATGGAGAGCATTGTGCTC, R)  followed by sequencing using LW23 |
| *sup-17(n316)*:  JKL1226 (AGTGAGGTCTTCTTCTGGAG, F)  JKL1227 (CGGTGCTTTCTCCAGAATTC, R)  followed by sequencing using JKL1226 |
| *tsp-12(ok239)*:  JKL1223 (TGTGCTGCCATGTGGCTTTC, F)  JKL1225 (GGTTGCATTTGCATGCATGC, R)  ZL210 (TTATCAGAGTAGAATCAGGGCTCCC, R)  or  LW47 (GTTTCGAGCAGTTTTACGCCACC, R)  LW49 (GGAATCAACGGAGCCGACGAT, R)  LW50 (AAGTTTCGGCAGACATCCTTCCG, F) |
| *tsp-14(jj95, jj96, jj97)*:  ZL178 (CGCTTGTGACTGGGAACA, R)  ZL179 (GACACACCGAGATACACTGAAA, F)  ZL180 (CAGAAGGACACGCGCTTTAT, F) |
| *sma-9(cc604)*:  MLF69(CGCAACAAGTTCATTCTCCA, F)  MLF70(CTTGGCTAAGATCCCATGCT, R)  followed by sequencing using MLF69 |
| *lin-12(ok2215):*  JKL1372 (CTCGAGACAAAGATCTGGAAGC, F)  JKL1373 (GACCGTCGAATTCAAGCAAC, R)  JKL1374 (GTGTATCGATGTAGATGCCC, R) |
| *hop-1(ar179):*  JKL1348 (ACGGACTGTTCCATTCATACG, F)  JKL1349 (TTTGAGGCCTCCAGTGATTC, F)  JKL1350 (CTCCCATTCCTAACCGAATTG, R) |
| *sel-12(ok2078):*  JJH13 (GCATGGCTCTGGTTGTTTTT, F)  JJH14 (TGGCCACATAACAAGCGATA, R)  JJH15 (GGAGAGGCGAGTTGTTCATC, F) |
| *sel-12(ar171)*:  JJH13 (GCATGGCTCTGGTTGTTTTT, F),  JJH16 (ATGGTCCTTTTGGTGTGAGC, R),  followed by sequencing using JKL1244 |
| *unc-40(e1430)*:  ZL49 (TTCCCAAGAGACCAGGGAGTTA, F),  ZL50 (AGAATCAGTGAGCAGTGCCGA, R),  followed by sequencing using ZL50 |
| *unc-40(ev495)* and *unc-40(tr115):*  LW11 (CAGGTCATGGCCCATTCTCTT, F),  LW12 (GTGTAACACTTACCACCAGCTCT, R),  followed by sequencing using LW11 |
|  |
| **Oligonucleotides for sgRNA plasmid construction used in CRISPR/Cas9-mediated genome editing** |
| For pZL8 (*tsp-14* knockout #1 sgRNA):  LW1 (CGGGAATTCCTCCAAGAACTCGTACAAAAATGCTCT)  ZL28 (ACGCGAGCTCGCGCGGTGGTCAAACATTTAGATTTGCAATTCAATTATATAG)  LW2 (CGGAAGCTTCACAGCCGACTATGTTTGGCGT)  ZL27 (GACCACCGCGCGAGCTCGCGTGTTTTAGAGCTAGAAATAGCAAGTTA) |
| For pZL9 (*tsp-14* knockout #2 sgRNA):  LW1 (CGGGAATTCCTCCAAGAACTCGTACAAAAATGCTCT)  ZL30 (CGGATGCCTTCAGCCGCTTCAAACATTTAGATTTGCAATTCAATTATATAG)  LW2 (CGGAAGCTTCACAGCCGACTATGTTTGGCGT)  ZL27 (GAAGCGGCTGAAGGCATCCGGTTTTAGAGCTAGAAATAGCAAGTTA) |
| For pLW4 (*sup-17::gfp* knockin #1 sgRNA):  LW1(CGGGAATTCCTCCAAGAACTCGTACAAAAATGCTCT)  LW13(GGCTCGAGGACAATGACTGCGTTTTAGAGCTAGAAATAGCAAGTTA)  LW2(CGGAAGCTTCACAGCCGACTATGTTTGGCGT)  LW14(GCAGTCATTGTCCTCGAGCCAAACATTTAGATTTGCAATTCAATTATATAG) |
| For pLW5 (*sup-17::gfp* knockin #2 sgRNA):  LW1(CGGGAATTCCTCCAAGAACTCGTACAAAAATGCTCT)  LW15(GTATACGGTGGTGGAGGCTCGGTTTTAGAGCTAGAAATAGCAAGTTA)  LW2(CGGAAGCTTCACAGCCGACTATGTTTGGCGT)  LW16(CGAGCCTCCACCACCGTATACAAACATTTAGATTTGCAATTCAATTATATAG) |
| For pZL57 (*tsp-12::gfp::3×flag* knockin #1 sgRNA):  ZL253 (TCTTGTCGGACATACTCGCTCAAC)  ZL254 (AAACTCTGATGGGTGAGAAGTGGC) |
| For pZL58 (*tsp-12::gfp::3×flag* knockin #2 sgRNA):  ZL255 (TCTTGACTCGCTCAACGGGCAAAA)  ZL256 (AAACTTTTGCCCGTTGAGCGAGTC) |
| For pZL60 (*gfp::3×flag::tsp-12* knockin #1 sgRNA):  ZL267 (TCTTGCCACTTCTCACCCATCAGA)  ZL268 (AAACTCTGATGGGTGAGAAGTGGC) |
| For pZL79 (*gfp::3×flag::tsp-12* knockin #2 sgRNA):  ZL334(TCTTGATGGCCAATCGACGACAGC)  ZL335(AAACGCTGTCGTCGATTGGCCATC) |
|  |
| **Oligonucleotides used to construct repair template for CRISPR/Cas9-mediated homologous recombination** |
| For pZL64 (*gfp::3×flag::tsp-12* knockin):  ZL283 (ACGTTGTAAAACGACGGCCAGTCGCCGGCAACCTCCCGTCGCTTTGTTCA)  ZL284 (TCCAGTGAACAATTCTTCTCCTTTACTCATCTGATGGGTGAGAAGTGGCCGA)  ZL285 (CGTGATTACAAGGATGACGATGACAAGAGAATGGCCAATCGACGACAGCCA  GTGCAACACAGAGCACAGCAGAG)  ZL286 (TCACACAGGAAACAGCTATGACCATGTTATGCTGAGAATTCGGCGATGAGC) |
| For pZL78 (*tsp-12::gfp::3×flag* knockin):  ZL287 (ACGTTGTAAAACGACGGCCAGTCGCCGGCATGAACGATGTGGTAG  TTGGATATAGAG)  ZL356 (CATCGATGCTCCTGAGGCTCCCGATGCTCCATGGGTATAATACCAC  TTAGCACGCTGGGCAAGGATGTCCGATTTGAGATTTTGAGCGAAACAG)  ZL289 (CGTGATTACAAGGATGACGATGACAAGAGATAATCAATCAAATAA  TCAATTAATCAACGAAA)  ZL290 (GGAAACAGCTATGACCATGTTATCGATTTCGGGCGTTCCGAGTGTA  CATAATTG) |
|  |
| **Oligonucleotides used to screen for successful genome editing events generated by CRISPR/Cas9** |
| *tsp-14(jj95, jj96, jj97)*:  ZL4 (CCCGCGGTGAGAAATGATGT, F)  ZL5 (TGCCGTCACTCATAACACCC, R) |
| *sup-17::gfp (jj98, jj99, jj100)*:  LW27 (ATCCAGGATCAGCAGCTGTA, R)  LW30 (AAGTGTTGCGCTGTACACAC, F)  JKL-269 (AAGCGTTCAACTAGCAGACC, F) |
| *gfp::3×flag::tsp-12(jj181, jj182):*  ZL349 (CGCCGGAATCACCCACGGAATGG, F)  ZL374 (CCCTCTGCTGTGCTCTGTGTTGC, F)  ZL375 (CTGAAGGGGAACATTTCTTTCCTGG, R) |
| *tsp-12::gfp::3×flag(jj194, jj196)*:  ZL349 (CGCCGGAATCACCCACGGAATGG, F)  ZL240 (AGGTTCTCGGTATCTGTTTCGCTC, R)  ZL242 (GGGCGTTCCGAGTGTACATAATTG, F) |
