# Supplementary material for: Two Paralogous Tetraspanins TSP-12 and TSP-14 Function with the ADAM10 Metalloprotease SUP-17 to Promote BMP Signaling in Caenorhabditis elegans
Source: PLoS Genet. 2017 Jan 9;13(1):e1006568. doi: 10.1371/journal.pgen.1006568 (PMC5261805; doi:10.1371/journal.pgen.1006568)
Supplement: S3 Table — (DOCX) [file pgen.1006568.s006.docx]

**Table S3. Plasmid constructs generated in this study.**

| **Plasmid name** | **Construct information** |
| --- | --- |
| **Tissue specific expression of *tsp-12 cDNA*** | |
| pJKL1080 | *hlh-8p::tsp-12 cDNA::unc-54 3’UTR* |
| pJKL1081 | *myo-2p::tsp-12 cDNA::unc-54 3’UTR* |
| pJKL1082 | *rol-6p::tsp-12 cDNA::unc-54 3’UTR* |
| pJKL1083 | *elt-3p::tsp-12 cDNA::unc-54 3’UTR* |
| pJKL1084 | *elt-2p::tsp-12 cDNA::unc-54 3’UTR* |
| pZL63 | *rab-3p::tsp-12 cDNA::unc-54 3’UTR* |
|  | |
| **Tissue specific expression of *sup-17 cDNA*** | |
| pJKL1036 | *rol-6p::sup-17 cDNA::sup-17 3’UTR* |
| pJKL1037 | *elt-2p::sup-17 cDNA::sup-17 3’UTR* |
| pJKL1038 | *hlh-8p::sup-17 cDNA::sup-17 3’UTR* |
| pJKL1140 | *sup-17p::sup-17 cDNA::sup-17 3’UTR* |
| pLW6 | *rab-3p::sup-17 cDNA::sup-17 3’UTR* |
|  | |
| **Translational reporters** | |
| pZL51 | *5kb tsp-12p::tsp-12 gDNA::gfp::1.7kb tsp-12 3’UTR* |
| pJKL1034 | *sup-17p::sup-17 gDNA::gfp::sup-17 3’UTR* |
|  | |
| **sgRNA constructs for CRISPR/Cas9 experiments** | |
| pZL8 | *tsp-14* knockout sgRNA plasmid #1 |
| pZL9 | *tsp-14* knockout sgRNA plasmid #2 |
| pZL57 | *tsp-12::gfp::3×flag* knockin sgRNA plasmid #1 |
| pZL58 | *tsp-12::gfp::3×flag* knockin sgRNA plasmid #2 |
| pZL60 | *gfp::3×flag::tsp-12* knockin sgRNA plasmid #1 |
| pZL79 | *gfp::3×flag::tsp-12* knockin sgRNA plasmid #2 |
| pLW4 | *sup-17* knockin sgRNA plasmid #1 |
| pLW5 | *sup-17* knockin sgRNA plasmid #2 |
|  | |
| **Repair templates for CRISPR/Cas9-mediated GFP knock-ins** | |
| pZL64 | *gfp::3×flag::tsp-12* |
| pZL78 | *tsp-12::gfp::3×flag* |
| pJKL1034 | *sup-17p::sup-17 gDNA::gfp::sup-17 3’UTR* |
|  | |
| **Constructs for the split-ubiquitin yeast two-hybrid experiments** | |
| pNAS27 | NubG(32)-TSP-12 ORF (described in [1]) |
| pNAS30 | NubG(32)-TSP-14 ORF (described in [1]) |
| pJKL1062 | NubG(32)-PAR-4 ORF (described in [1]) |
| pNAS22 | SUP-17 ORF-CubPLV |

1. Liu Z, Shi H, Szymczak LC, Aydin T, Yun S, Constas K, et al. Promotion of bone morphogenetic protein signaling by tetraspanins and glycosphingolipids. PLoS Genet. 2015;11(5):e1005221.
